# Supplementary material for: Mitogenomics of Chinch Bugs from China and Implications for Its Coevolutionary Relationship with Grasses
Source: Insects. 2022 Jul 17;13(7):643. doi: 10.3390/insects13070643 (PMC9315831; doi:10.3390/insects13070643)
Supplement: Supplementary file 1 [file insects-13-00643-s001.zip › insects-1798814-supplementary.pdf]

Supplementary Materials for

Mitogenomics of the chinch bugs from China and implications for its coevolutionary relationship with grasses

Shujing Wang[a], Runqi Zhu[a], Huaijun Xue[a], Yanfei Li [a]*, Wenjun Bu[a]*

a. Institute of Entomology, College of Life Sciences, Nankai University, Tianjin 300071, PR China

*Corresponding author.

E-mail address: wenjunbu@nankai.edu.cn (W.-J. Bu); liyanfei9412@163.com (Y.-F. Li)

TABLE S1. Experimental sample information of Blissidae.

Number	species	Sample_ID	Latitude	Longitude
1	*Bochrus foveatus*	BoYNGL1	24.0969	97.8262
2	*Capodemus sinuatus*	CpYNRL1	24.0969	97.8262
3	*Cavelerius yunnanensis*	CvYNLC1	23.0553	102.5331
4	*Dimorphopterus gibbus*	DmGXBS1	24.2790	106.2195
5	*Iphicrates gressitti*	IpZJLA1	30.1153	118.9892
6	*Ischnodemus noctulus*	IsYNRL1	24.0969	97.8262
7	*Macropes harringtonae*	MaGZZY1	27.6738	107.2768
8	*Macropes dentipes*	MaYNBN1	22.1100	100.3640
9	*Macropes robustus*	MaYNGL1	24.0969	97.8262
10	*Pirkimerus japonicus*	PiGXBS1	24.3890	106.4065

TABLE S2. Public data used in phylogenetic analysis.

Superfamily	Species	Accession number
Lygaeoidea	*Metatropis longirostris*	NC_037373
Lygaeoidea	*Yemmalysus parallelus*	NC_012464
Lygaeoidea	*Phaenacantha marcida*	NC_012460
Lygaeoidea	*Neolethaeus assamensis*	NC_037375
Pyrrhocoroidea	*Physopelta cincticollis*	NC_042433
Pyrrhocoroidea	*Physopelta gutta*	NC_012432
Pyrrhocoroidea	*Antilochus coquebertii*	NC_042441
Pyrrhocoroidea	*Antilochus russus*	NC_042440

TABLE S3. Best-fit models of sequence evolution and partitioning schemes selected by PartitionFinder for phylogenetic reconstructions.

Subset	Best model	Subset partitions
		**Subset Best partitioning scheme for Mrbayes (LnL= -79323.7575125)**
1	GTR+I+G	ND2, ND6, ATPase8, ND3,ATPase6,rRNA,tRNA
2	GTR+I+G	CO1, CytB, CO2
3	K81UF+I+G	ND4L, ND1, ND4, ND5

TABLE S4. The nonsynonymous nucleotide changes (Ka) and the synonymous nucleotide changes (Ks) of PCGs.

	Ks	Ka	Ka/Ks
ATP6	0.4439	0.1269	0.2859
ATP8	0.5206	0.2732	0.5249
COI	0.4647	0.0557	0.1198
COII	0.4796	0.0903	0.1882
COIII	0.4832	0.1133	0.2345
CYTB	0.5352	0.0996	0.1860
ND1	0.3586	0.1160	0.3235
ND2	0.4689	0.2136	0.4556
ND3	0.4905	0.1324	0.2699
ND4	0.3926	0.1732	0.4411
ND4L	0.3741	0.1589	0.4246
ND5	0.3410	0.1913	0.5609
ND6	0.5084	0.2588	0.5090
